# An easy microwave-assisted synthesis of *C*8-alkynyl adenine pyranonucleosides as novel cytotoxic antitumor agents

**DOI:** 10.3389/fchem.2015.00021

**Published:** 2015-03-23

**Authors:** Athina Dimopoulou, Stella Manta, Vanessa Parmenopoulou, Nikolaos Kollatos, Ourania Christidou, Virginia V. Triantakonstanti, Dominique Schols, Dimitri Komiotis

**Affiliations:** ^1^Laboratory of Bioorganic Chemistry, Department of Biochemistry and Biotechnology, University of ThessalyLarissa, Greece; ^2^Laboratory of Organic Chemistry, Department of Chemistry, Aristotle University of ThessalonikiThessaloniki, Greece; ^3^Department of Microbiology and Immunology, Rega Institute for Medical Research, KU LeuvenLeuven, Belgium

**Keywords:** Sonogashira coupling reaction, 8-bromoadenine, *N*^6^-benzoyladenine pyranonucleosides, microwave irradiation, cytotoxic activity

## Abstract

We describe the synthesis of *C*8-alkynyl adenine pyranonucleosides **4, 5**, and 8-phenylethynyl-adenine **(II)**, *via* Sonogashira cross-coupling reaction under microwave irradiation. Compounds **4e** and **II** were less cytostatic than 5-fluorouracil (almost an order of magnitude) against murine leukemia (L1210) and human cervix carcinoma (HeLa) cells, while the same compounds proved to be more active than 5-fluorouracil against human lymphocyte (CEM) cells.

## Introduction

Synthetic nucleoside analogs with modified nucleobase moieties are of considerable importance in the search for promising lead candidates endowed with antiviral, anticancer, and antibacterial activities (Herdewijn, 2008; Manta et al., 2014). Among them, a number of purine and pyrimidine substituted nucleoside derivatives exhibited activity in both solid tumors and hematological malignancies, behaving as antimetabolites, competing with physiological nucleosides, and consequently, interacting with a large number of intracellular targets to induce cytotoxicity (Hatse et al., 1999).

Alkynyl-modified nucleosides and especially pyrimidine derivatives substituted at *C*5 and purine derivatives substituted at *C*8, have been shown to possess interesting biological properties (Lin et al., 1985; Meneni et al., 2007; Lee et al., 2009; Vivet-Boudou et al., 2011). Some representative examples include 5-ethynyl-2′-deoxyuridine, which exhibited antiproliferative activity against human breast cancer cells, exceeding that of cisplatin and 5-fluorouracil, while 5-bromoethynyluridine demonstrated significant anti-HCV properties (Escuret et al., 2005; Meneni et al., 2007). Although, little effort has been made toward the synthesis of *C*8-modified purine nucleosides, in some cases, interesting biological properties have been reported, such as some 8-alkynyl adenosines, which proved to be very selective ligands for the A_3_ adenosine receptor subtype behaving as adenosine antagonists (Volpini et al., 2001) and various *C*8-modified 2′-deoxy adenosines, which induced delayed chain termination *in vitro* and showed moderate anti HIV-1 activity in cell culture (Vivet-Boudou et al., 2011).

Considering all the progress made toward this direction, we have recently embarked on the synthesis of *C*5-substituted uracil and cytosine glucopyranonucleosides bearing a variety of alkyne substituents, such as linear alkyl chains and aromatic rings substituted with linear and branched alkyl groups (Dimopoulou et al., 2013), which effectively inhibited the proliferation of a variety of tumor cell lines and they also proved as some of the most potent inhibitors of the active site of glycogen phosphorylase (Kantsadi et al., 2012). Among these agents, the *C*5-phenylethynyluracil pyranonucleoside showed appreciable cytotoxic activity (IC_50_ of 5.2–6.2 μM), comparable to 5-fluorouracil (Dimopoulou et al., 2013).

As a continuation of our studies on the synthesis of base-modified pyranonucleosides and considering the interesting biological properties of substituted purines, it was speculated that the introduction of alkynyl modifications at the 8-position of either adenine pyranonucleosides or even adenine itself, could possibly lead to more efficacious therapeutic agents. This conjugation appeared to us as a challenge and the first biological results confirmed our hypothesis.

## Experimental (general methods)

Melting points were recorded in a Mel-Temp apparatus and are uncorrected. Thin layer chromatography (TLC) was performed on Merck precoated 60F254 plates. Reactions were monitored by TLC on silica gel, with detection by UV light (254 nm) or by charring with sulfuric acid. Flash column chromatography was performed using silica gel (240–400 mesh, Merck). ^1^H and ^13^C NMR spectra were obtained at room temperature with a Bruker 300 spectrometer at 300 and 75.5 MHz, respectively using chloroform-*d* (CDCl_3_) and dimethylsulfoxide-*d*_6_ (DMSO-*d*_6_) with internal tetramethylsilane (TMS). The ^1^H assignments were based on ^1^H -^1^H COSY experiments executed using standard Varian software. Chemical shifts (δ) were given in ppm measured downfield from TMS, and spin-spin coupling constants are in Hz. Mass spectra were obtained on a ThermoQuest Finnigan AQA Mass Spectrometer (electrospray ionization). Optical rotations were measured using an Autopol I polarimeter and UV-Vis spectra were recorded on a PG T70 UV-VIS spectrometer. Acetonitrile (CH_3_CN) was distilled from calcium hydride and stored over 3Å molecular sieves. *N,N*-Dimethylformamide (DMF) was stored over 3Å molecular sieves. All reactions sensitive to oxygen or moisture were carried out under nitrogen atmosphere using oven-dried glassware. All microwave irradiation experiments were carried out in a dedicated CEM-Explorer and CEM Discover monomode microwave apparatus, operating at a frequency of 2.45 GHz with continuous irradiation power from 0 to 300 W with utilization of the standard absorbance level of 300 W maximum power. The reactions were carried out in 10-mL glass tubes, sealed with a Teflon septum and placed in the microwave cavity. Initially, microwave irradiation of required watts was used, and the temperature was ramped from room temperature to the desired temperature. Once this was reached the reaction mixture was held at this temperature for the required time. The reaction mixture was continuously stirred during the reaction. The temperature was measured with an IR sensor on the outer surface of the process vial. After the irradiation period, gas jet cooling rapidly cooled the reaction vessel to ambient temperature.

### 9-(2′,3′,4′,6′-tetra-*O*-acetyl-β-*D*-glucopyranosyl)-*N*^6^-benzoyl adenine (2)

A mixture of *N*^6^-benzoyladenine (797 mg, 3.33 mmol, 1.3 equiv), hexamethyldisilazane (HMDS) (871 μL, 4.13 mmol, 1.24 equiv) and saccharine (27 mg, 0.15 mmol, 0.046 equiv) in anhydrous CH_3_CN (14 mL) was refluxed for 1 h under nitrogen. 1,2,3,4,6-Penta-*O*-acetyl-*D*-glucopyranose **(1)** (1 g, 2.56 mmol) and tin(IV) chloride (SnCl_4_) (419 μL, 3.58 mmol, 1.4 equiv) were then added and the reaction mixture was stirring under reflux for 2 more h, cooled, neutralized with aqueous sodium bicarbonate, and extracted with ethyl acetate (1000 mL). The organic extract was dried over anhydrous sodium sulfate, filtered, and evaporated to dryness. The residue was purified by flash column chromatography (EtOAc/ hexane 7:3) to give compound **2**, as a white solid (814 mg, 56%); mp 169–171°C; [α]^22^_D_ = −6 (c 0.2, CHCl_3_); *R*_f_ = 0.47 (EtOAc); λ_max_ 280 nm (ε 20557); ^1^H NMR (CDCl_3_,300 MHz): δ 8.83 (s, 1H, H-2), 8.22 (s, 1H, H-8), 8.04-7.51 (m, 5H, Bz), 5.98 (d, 1H, *J*_1′,2′_ = 9.4 Hz, H-1′), 5.65 (t, 1H, *J* = 9.3 Hz, H-3′), 5.50 (t, 1H, *J* = 9.4 Hz, H-2′), 5.32 (t, 1H, *J* = 9.7 Hz, H-4′), 4.34 (dd, 1H, *J*_5′,6a′_ = 4.1 Hz, *J*_6a′,6b′_ = 12.8 Hz, H-6a′), 4.24-4.14 (m, 1H, H-6b′), 4.11-4.02 (m, 1H, H-5′), 2.08, 2.07, 2.04, 1.79 (4s, 12H, 4OAc);^13^C NMR (CDCl_3_, 75.5 MHz): δ 170.4, 169.8, 169.3, 169.0, 164.5, 152.0, 151.9, 149.5, 140.9, 133.1, 133.0, 128.9, 128.1, 122.2, 80.6, 75.2, 72.7, 70.3, 67.7, 61.5, 20.6, 20.5, 20.4, 20.1; Mass (M+H)^+^: 570.16; Anal. Calcd. for C_26_H_27_N_5_O_10_: C, 54.83; H, 4.78; N, 12.30%. Found: C, 55.16; H, 4.66; N, 12.53%.

### 9-(2′,3′,4′,6′-tetra-*O*-acetyl-β-*D*-glucopyranosyl)- 8-bromo-*N*^6^-benzoyl adenine (3)

To a solution of 9-(2′,3′,4′,6′-tetra-*O*-acetyl-*β-D*-glucopyranosyl)-*N*^6^-benzoyl adenine **(2)** (250 mg, 0.44 mmol), and sodium acetate (172 mg, 2.1 mmol) in 1.6 mL of glacial acetic acid, 75 μL, 1.45 mmol of bromine were added. The reaction was left to stir at room temperature until completion (14 h). The whole was extracted with ethyl acetate and satured Na_2_S_2_O_3_. The organic layer was washed with water and brine, dried over anhydrous sodium sulfate, filtered, and evaporated to dryness. The residue was purified by flash column chromatography (EtOAc/ hexane 7:3) to give compound **3**, (171 mg, 60%); [α]^22^_D_ = −3 (c 0.2, CHCl_3_); *R*_f_ = 0.45 (EtOAc/ hexane 7:3); λ_max_ 282 nm (ε 21223); ^1^H NMR (CDCl_3_,300 MHz): δ 8.82 (s, 1H, H-2), 8.04-7.49 (m, 5H, Bz), 6.21 (t, 1H, *J* = 9.6 Hz, H-2′), 5.96 (d, 1H, *J*_1′,2′_ = 9.6 Hz, H-1′), 5.47 (t, 1H, *J* = 9.4 Hz, H-3′), 5.35 (t, 1H, *J* = 9.9 Hz, H-4′), 4.26-4.25 (m, 2H, H-6a′, H-6b′), 4.06-3.96 (m, 1H, H-5′), 2.09, 2.07, 2.04, 1.79 (4s, 12H, 4OAc);^13^C NMR (CDCl_3_, 75.5 MHz): δ 170.5, 170.0, 169.9, 169.3, 164.7, 153.3, 152.9, 149.2, 133.3, 131.0, 128.9, 128.6, 128.2, 122.3, 78.2, 72.9, 68.8, 67.6, 67.5, 61.5, 20.7, 20.6, 20.5, 20.2; Mass (M+H)^+^: 648.08; Anal. Calcd. for C_26_H_26_BrN_5_O_10_: C, 48.16; H, 4.04; Br, 12.32; N, 10.80%. Found: C, 48.44; H, 4.15; Br, 12.11; N, 10.99%.

## General experimental procedure for the preparation of protected *C*8-alkynyl adenine pyranonucleosides (4a–e)

The appropriate alkynes (3 equiv), Pd(PPh_3_)_4_ (43 mg, 0.1 equiv), CuI (7 mg, 0.1 equiv), triethylamine (103 μL, 2 equiv) and 9-(2′,3′,4′,6′-tetra-*O*-acetyl-β-*D*-glucopyranosyl)-8-bromo-*N*^6^-benzoyl adenine **(3)** (240 mg, 0.37 mmol), were irradiated under microwaves (200 W) in 1 mL of anhydrous DMF for 10 min at 120°C. The reaction mixture was concentrated under reduced pressure and the crude residue was purified by flash chromatography on silica gel. The purified material was dried *in vacuo* to afford the corresponding derivatives **4a–e**, as colorless foams.

### 9-(2′,3′,4′,6′-tetra-*O*-acetyl-β-*D*-glucopyranosyl)-8-heptynyl-*N*^6^-benzoyl adenine (4a)

139 mg, 56%; [α]^22^_D_ = −4 (c 0.4, CHCl_3_); *R*_f_ = 0.10 (EtOAc/ hexane 6:4); λ_max_ 303 nm (ε 22594); ^1^H NMR (CDCl_3_,300 MHz): δ 8.99 (br s, 1H, NH), 8.85 (s, 1H, H-2), 8.03-7.41 (m, 5H, Bz), 6.28 (t, 1H, *J* = 9.4 Hz, H-2′), 5.95 (d, 1H, *J*_1′,2′_ = 9.2 Hz, H-1′), 5.46 (t, 1H, *J* = 9.3 Hz, H-3′), 5.35 (t, 1H, *J* = 9.3 Hz, H-4′), 4.28-4.19 (m, 2H, H-6a′, H-6b′), 4.04-3.87 (m, 1H, H-5′), 2.62 (t, 2H, *J* = 7.1 Hz, α-CH_2_), 2.09, 2.08, 2.05, 1.78 (4s, 12H, 4OAc), 1.56-1.35 (m, 6H, 3 × CH_2_), 0.96 (t, 3H, *J* = 7.2 Hz, CH_3_);^13^C NMR (CDCl_3_, 75.5 MHz): δ 170.8, 170.5, 170.0, 169.8, 165.0, 153.5, 152.2, 151.3, 148.8, 134.9, 133.0, 129.2, 127.9, 119.9, 97.7, 94.5, 82.9, 70.8, 70.1, 67.5, 67.2, 63.7, 31.5, 31.2, 29.6, 22.3, 21.5, 21.3, 21.0, 20.9, 15.2; Mass (M+H)^+^: 664.26; Anal. Calcd. for C_33_H_37_N_5_O_10_: C, 59.72; H, 5.62; N, 10.55%. Found: C, 59.96; H, 5.94; N, 10.65%.

### 9-(2′,3′,4′,6′-tetra-*O*-acetyl-β-*D*-glucopyranosyl)-8-phenylethynyl-*N*^6^-benzoyl adenine (4b)

161 mg, 66%; [α]^22^_D_ = −6 (c 0.4, CHCl_3_); *R*_f_ = 0.33 (CH_2_Cl_2_/ MeOH 9.8:0.2); λ_max_ 300 nm (ε 29706); ^1^H NMR (CDCl_3_, 300 MHz): δ 8.95 (br s, 1H, NH), 8.87 (s, 1H, H-2), 8.01-7.45 (m, 10H, Bz and Ph), 6.31 (t, 1H, *J* = 9.3 Hz, H-2′), 6.10 (d, 1H, *J*_1′,2′_ = 9.4 Hz, H-1′), 5.49 (t, 1H, *J* = 9.2 Hz, H-3′), 5.35 (t, 1H, *J* = 9.5 Hz, H-4′), 4.31-4.22 (m, 2H, H-6a′, H-6b′), 4.08-3.98 (m, 1H, H-5′), 2.09, 2.05, 1.90, 1.77 (4s, 12H, 4OAc);^13^C NMR (CDCl_3_, 75.5 MHz): δ 170.6, 170.3, 169.9, 169.7, 165.3, 153.1, 152.4, 150.5, 148.5, 134.5, 133.8, 133.5, 129.9, 129.5, 129.0, 128.3, 123.6, 119.3, 102.6, 98.7, 87.3, 83.6, 70.5, 67.9, 67.5, 63.5, 21.5, 21.3, 20.9, 20.5; Mass (M+H)^+^: 670.18; Anal. Calcd. for C_34_H_31_N_5_O_10_: C, 60.98; H, 4.67; N, 10.46%. Found: C, 61.23; H, 4.28; N, 10.80%.

### 9-(2′,3′,4′,6′-tetra-*O*-acetyl-β-*D*-glucopyranosyl)-8-p-tolylethynyl-*N*^6^-benzoyl adenine (4c)

157 mg, 61%; [α]^22^_D_ = −10 (c 0.3, CHCl_3_); *R*_f_ = 0.37 (EtOAc/ hexane 8:2); λ_max_ 325 nm (ε 27336); ^1^H NMR (CDCl_3_, 300 MHz): δ 8.98 (br s, 1H, NH), 8.86 (s, 1H, H-2), 8.02-7.29 (m, 9H, Bz and ArH), 6.31 (t, 1H, *J* = 9.4 Hz, H-2′), 6.08 (d, 1H, *J*_1′,2′_ = 9.3 Hz, H-1′), 5.48 (t, 1H, *J* = 9.4 Hz, H-3′), 5.37 (t, 1H,*J* = 9.7 Hz, H-4′), 4.29-4.22 (m, 2H, H-6a′, H-6b′), 4.07-3.98 (m, 1H, H-5′), 2.43 (s, 3H, CH_3_), 2.09, 2.05, 1.91, 1.77 (4s, 12H, 4OAc);^13^C NMR (CDCl_3_, 75.5 MHz): δ 170.3, 170.1, 169.7, 169.3, 165.1, 153.2, 152.3, 150.7, 148.7, 139.5, 135.8, 133.7, 133.4, 129.6, 129.2, 128.8, 120.3, 120.1, 102.4, 98.3, 87.6, 83.5, 70.7, 67.5, 67.1, 63.7, 22.5, 21.7, 21.5, 21.0, 20.2; Mass (M+H)^+^: 684.22; Anal. Calcd. for C_35_H_33_N_5_O_10_: C, 61.49; H, 4.87; N, 10.24%. Found: C, 61.84; H, 4.52; N, 10.02%.

### 9-(2′,3′,4′,6′-tetra-*O*-acetyl-β-*D*-glucopyranosyl)-8-(pyridin-3-yl-ethynyl)-*N*^6^-benzoyl adenine (4d)

141 mg, 58%; [α]^22^_D_ = −12 (c 0.2, CHCl_3_); *R*_f_ = 0.23 (EtOAc/ hexane 6:4); λ_max_ 320 nm (ε 16156); ^1^H NMR (CDCl_3_, 300 MHz): δ 8.99 (br s, 1H, NH), 8.87 (s, 1H, H-2), 8.72-7.39 (m, 9H, Bz and pyridine), 6.26 (t, 1H, *J* = 9.5 Hz, H-2′), 6.11 (d, 1H, *J*_1′,2′_ = 9.3 Hz, H-1′), 5.48 (t, 1H, *J* = 9.3 Hz, H-3′), 5.35 (t, 1H, *J* = 9.7 Hz, H-4′), 4.32 (dd, 1H,*J*_5′,6a′_ = 4.7 Hz, *J*_6a′,6b′_ = 12.5 Hz, H-6a′), 4.21 (dd, 1H,*J*_5′,6b′_ = 2.0 Hz, J _6a′,6b′_ = 12.5 Hz, H-6b′), 4.09-4.00 (m, 1H, H-5′), 2.09, 2.05, 1.94, 1.78 (4s, 12H, 4OAc);^13^C NMR (CDCl_3_, 75.5 MHz): δ 170.4, 170.0, 169.6, 169.2, 165.4, 153.0, 152.7, 151.6, 150.7, 150.5, 148.8, 140.5, 135.4, 133.6, 129.5, 128.2, 124.6, 120.5, 117.1, 98.7, 95.6, 83.3, 75.7, 70.5, 67.5, 67.2, 63.5, 21.3, 21.0, 20.9, 20.7; Mass (M+H)^+^: 671.21; Anal. Calcd. for C_33_H_30_N_6_O_10_: C, 59.10; H, 4.51; N, 12.53%. Found: C, 59.42; H, 4.76; N, 12.22%.

### 9-(2′,3′,4′,6′-tetra-*O*-acetyl-β-*D*-glucopyranosyl)-8-(pyridin-2-yl-ethynyl)-*N*^6^-benzoyl adenine (4e)

127 mg, 50%; [α]^22^_D_ = −2 (c 0.1, CHCl_3_); *R*_f_ = 0.21 (EtOAc/ hexane 7:3); λ_max_ 315 nm (ε 22103); ^1^H NMR (CDCl_3_, 300 MHz): δ 8.80 (br s, 2H, NH, H-2), 8.07-7.35 (m, 9H, Bz and pyridine), 6.31 (t, 1H,*J* = 9.4 Hz, H-2′), 6.12 (d, 1H, *J*_1′,2′_ = 9.3 Hz, H-1′), 5.71 (t, 1H, *J* = 9.5 Hz, H-3′), 5.48 (t, 1H, J = 9.5 Hz, H-4′), 4.29-4.25 (m, 2H, H-6a′, H-6b′), 4.10-4.02 (m, 1H, H-5′), 2.09, 2.03, 1.92, 1.75 (4s, 12H, 4OAc);^13^C NMR (CDCl_3_, 75.5 MHz): δ 170.6, 170.3, 169.7, 169.5, 164.3, 153.2, 152.8, 151.5, 149.9, 148.3, 145.2, 139.5, 134.6, 132.7, 128.6, 127.8, 127.6, 123.7, 119.7, 95.7, 92.5, 84.8, 82.7, 72.8, 69.5, 68.3, 65.7, 21.0, 20.7, 20.6, 20.5; Mass (M+H)^+^: 671.20; Anal. Calcd. for C_33_H_30_N_6_O_10_: C, 59.10; H, 4.51; N, 12.53%. Found: C, 59.40; H, 4.73; N, 12.34%.

## General procedure for the preparation of unprotected *C*8-alkynyl adenine pyranonucleosides (5a–d)

The protected nucleosides **4a–e** (0.5 mmol), were treated with methanolic ammonia (satured at 0°C, 27.9 mL). The solution was stirred overnight at room temperature and then evaporated under reduced pressure. The residue was purified by flash column chromatography to afford the unprotected derivatives **5a–d**, in 60–72% yields, as yellowish and white foams.

### 9-(β-*D*-glucopyranosyl)-8-heptynyl-adenine (5a)

117 mg, 60%; [α]^22^_D_ = −2 (c 0.2, MeOH); *R*_f_ = 0.17 (EtOAc/ MeOH 8:2) λ_max_ 292 nm (ε 19971); ^1^H NMR (DMSO-*d*_6_, 300 MHz): δ 8.15 (s, 1H, H-2), 7.24 (s, 2H, NH_2_), 5.43 (d, 1H, *J*_1′,2′_ = 9.5 Hz, H-1′), 5.16, 5.09, 5.00 (3br s, 3H, 3OH), 4.60-4.45 (m, 2H, OH, H-2′), 3.81-3.34 (m, 5H, H-3′, H-4′, H-5′, H-6a′, H-6b′), 1.78-1.76 (m, 2H, β-CH_2_), 1.63 (t, 2H, *J* = 7.2 Hz, α-CH_2_), 1.51-1.30 (m, 4H, 2 × CH_2_), 0.93 (t, 3H, J = 7.2 Hz, CH_3_). ^13^C NMR (DMSO-*d*_6_, 75.5 MHz): δ 156.8, 152.7, 150.1, 148.3, 119.9, 96.2, 93.9, 83.2, 78.8, 73.6, 70.5, 66.7, 65.4, 30.9, 29.2, 22.3, 19.0, 14.5; Mass (M+H)^+^: 392.20; Anal. Calcd. for C_18_H_25_N_5_O_5_: C, 55.23; H, 6.44; N, 17.89%. Found: C, 54.90; H, 6.73; N, 17.72%.

### 9-(β-*D*-glucopyranosyl)-8-phenylethynyl-adenine (5b)

143 mg, 72%; [α]^22^_D_ = -6 (c 0.1, MeOH); R_f_ = 0.30 (EtOAc/ MeOH 9:1); λ_max_ 313 nm (ε 14209); ^1^H NMR (DMSO-*d*_6_, 300 MHz): δ 8.19 (s, 1H, H-2), 7.66 (br s, 2H, NH_2_), 7.61-7.47 (m, 5H, Ph), 5.55 (d, 1H, *J*_1′,2′_ = 9.2 Hz, H-1′), 5.40 (d, 1H, *J* = 5.3 Hz, OH), 5.34 (d, 1H, *J* = 4.1 Hz, OH), 5.21 (d, 1H, *J* = 5.1 Hz, OH), 4.70 (t, 1H, *J*= 4.4 Hz, OH), 4.54-4.40 (m, 1H, H-2′), 3.81-3.39 (m, 5H, H-3′, H-4′, H-5′, H-6a′, H-6b′). ^13^C NMR (DMSO-*d*_6_, 75.5 MHz): δ 156.3, 152.6, 150.0, 148.1, 132.7, 128.9, 128.7, 123.0, 119.7, 101.9, 96.3, 86.8, 83.3, 78.1, 73.4, 70.8, 65.8; Mass (M+H)^+^: 398.15; Anal. Calcd. for C_19_H_19_N_5_O_5_: C, 57.43; H, 4.82; N, 17.62%. Found: C, 57.81; H, 4.51; N, 17.90%.

### 9-(β-*D*-glucopyranosyl)-8-*p*-tolylethynyl-adenine (5c)

140 mg, 67%; [α]^22^_D_ = −4 (c 0.1, MeOH); *R*_f_ = 0.21 (EtOAc/ MeOH 8:2); λ_max_ 316 nm (ε 12871); ^1^H NMR (DMSO-*d*_6_, 300 MHz): δ 8.18 (s, 1H, H-2), 7.55 (d, 2H, J = 8.1 Hz, ArH), 7.37 (br s, 2H, NH_2_), 7.32 (d, 2H, *J* = 8.0 Hz, ArH), 5.55 (d, 1H, *J*_1′,2′_ = 9.3 Hz, H-1′), 5.25 (d, 1H, *J*= 4.1 Hz, OH), 5.15, 5.04 (2br s, 2H, 2OH), 4.58-4.47 (m, 2H, OH, H-2′), 3.81-3.34 (m, 5H, H-3′, H-4′, H-5′, H-6a′, H-6b′), 2.38 (s, 3H, CH_3_). ^13^C NMR (DMSO-*d*_6_, 75.5 MHz): δ 156.4, 152.1, 150.4, 148.5, 138.5, 132.5, 129.0, 122.3, 120.2, 101.8, 96.0, 86.9, 83.0, 78.9, 73.5, 70.7, 65.6, 24.6; Mass (M+H)^+^: 412.17; Anal. Calcd. for C_20_H_21_N_5_O_5_: C, 58.39; H, 5.14; N, 17.02%. Found: C, 58.72; H, 5.45; N, 17.27%.

### 9-(β-*D*-glucopyranosyl)-8-(pyridin-3-yl-ethynyl)-adenine (5d)

139 mg, 70%; [α]^22^_D_ = +2 (c 0.2, MeOH); *R*_f_ = 0.16 (CH_2_Cl_2_/ MeOH 7:3); λ_max_ 306 nm (ε 7353); ^1^H NMR (DMSO-*d*_6_, 300 MHz): δ 8.89-8.68 (m, 2H, pyridine), 8.21 (s, 1H, H-2), 8.10-7.52 (m, 2H, pyridine), 7.44 (br s, 2H, NH_2_), 5.58 (d, 1H, *J*_1′,2′_ = 9.3 Hz, H-1′), 5.28, 5.07, 4.56 (3 br s, 3H, 3OH), 4.45 (t, 1H,*J* = 8.1 Hz, OH), 3.81-3.40 (m, 6H, H-2′, H-3′, H-4′, H-5′, H-6a′, H-6b′). ^13^C NMR (DMSO-*d*_6_, 75.5 MHz): δ 156.9, 152.5, 152.3, 150.5, 149.8, 148.5, 139.9, 123.8, 119.8, 116.8, 96.0, 95.7, 83.1, 78.7, 74.1, 73.9, 70.2, 65.2; Mass (M+H)^+^: 399.15; Anal. Calcd. for C_18_H_18_N_6_O_5_: C, 54.27; H, 4.55; N, 21.10%. Found: C, 54.62; H, 4.74; N, 21.38%.

### 8-phenylethynyl-adenine (II)

8-Bromoadenine **(I)** (43 mg, 0.2 mmol) was mixed with anhydrous DMF (1 mL), phenylacetylene (66 μL, 3 equiv), triethylamine (55 μL, 2 equiv), Pd(PPh_3_)_4_ (23 mg, 0.1 equiv), CuI (11 mg, 0.3 equiv) and irradiated with microwaves (200 W) for 6 min at 60°C. After removing volatiles *in vacuo*, the solid residue was purified by flash chromatography (CH_2_Cl_2_/MeOH, 9.5:0.5) to provide compound **II**, as yellowish foam. 19 mg, 40%; [α]^22^_D_ = −2 (c 0.2, MeOH); *R*_f_ = 0.13 (CH_2_Cl_2_/ MeOH 8.5:1.5); λ_max_ 308 nm (ε 9701); ^1^H NMR (DMSO-*d*_6_, 300 MHz): δ 13.38 (br s, 1H, NH), 8.15 (s, 1H, H-2), 7.65-7.49 (m, 5H, Ph), 7.27 (br s, 2H, NH_2_). ^13^C NMR (DMSO-*d*_6_, 75.5 MHz): δ 156.8, 154.2, 152.6, 148.1, 132.7, 129.1, 129.0, 123.0, 119.8, 101.8, 67.1; Mass (M+H)^+^: 236.08; Anal. Calcd. for C_13_H_9_N_5_: C, 66.37; H, 3.86; N, 29.77%. Found: C, 66.59; H, 4.01; N, 29.40%.

### Antiproliferative assay

Compounds **4e**, **5a–d**, and **II**, were evaluated for their cytostatic activity against human cervix carcinoma (HeLa) cells, human lymphocytes (CEM) as well as murine leukemia (L1210) cells. All assays were performed in 96-well microtiter plates. To each well (5–7.5) × 10^4^ tumor cells were added and varying concentrations of the test compounds ranging from 250, 50, 10, 2, 0.4, to 0.08 μM. The tumor cells were allowed then to proliferate at 37°C in a humidified CO_2_-controlled atmosphere. To obtain their optimal growth curves this is for 2 days of the murine leukemia (L1210) cells and for 3 days for the human lymphocytic (CEM) cells and the human cervix carcinoma (HeLa) cells. At the end of the incubation period, the cells were counted in a Coulter counter. The IC_50_ (50% inhibitory concentration) was defined as the concentration of the compound that inhibited cell proliferation by 50%. Experiments were repeated at least three times and these data are presented in Table 1.

**Table 1:** **Cytostatic activity of the compounds against tumor cell (L1210, CEM, and HeLa) proliferation**.

**Compound**	**IC^a^_50_ (μM)**		
	**L1210**	**CEM**	**HeLa**
**4e**	2.9 ± 0.0	1.2 ± 0.2	3.0 ± 0.7
**5a**	>250	>250	>250
**5b**	>250	>250	>250
**5c**	>250	>250	>250
**5d**	>250	>250	≥250
**II**	5.9 ± 5.5	4.2 ± 0.8	10 ± 3.0
**5-Fluorouracil**	0.33 ± 0.17	18 ± 5	0.54 ± 0.12

a *50% inhibitory concentration or compound concentration required to inhibit tumor cell proliferation by 50%*.

## Results and discussion

**Figure 1:**
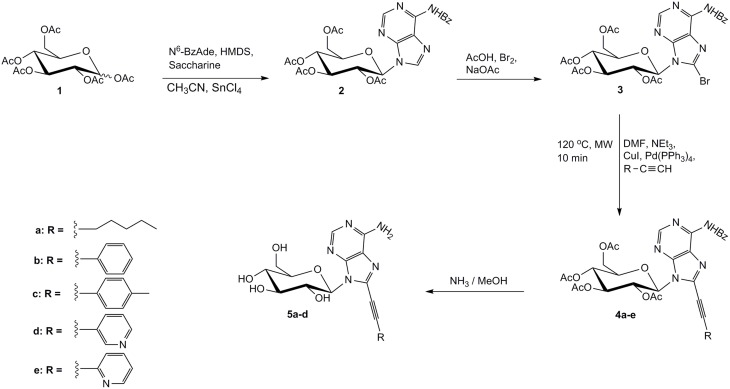
**Synthesis of *C*8-alkynyl adenine glucopyranonucleosides**.

Herein, we describe the synthesis of *C*8-alkynyl adenine pyranonucleosides and present their biological properties. The starting material of our synthesis was the commercially available per-*O*-acetylated D-glucose **1** which upon coupling, *via* two-step Vorbrüggen method (Vorbrüggen and Höfle, 1981), with *N*^6^-benzoyl adenine, gave selectively the *N*9-isomeric adenine nucleoside **2**, under thermodynamically controlled conditions (SnCl_4_/CH_3_CN, reflux). Trans rule was followed (Baker, 1957) and the β-configured nucleoside **2** was solely obtained as deduced from ^1^H NMR vicinal coupling data (*J*_1′,2′_ = 9.4 Hz). Since halogenated *C*8-purine nucleosides have proven useful intermediates for the efficient preparation of their corresponding *C*8-alkynyl derivatives (Agrofoglio et al., 2003), the adenine nucleoside **2** was directly brominated after treatment with Br_2_/AcOH/AcONa (Holmes and Robins, 1964), affording the 8-bromo analog **3**, in approximately 60% yield after aqueous work-up and flash chromatography. The position of substitution of the bromine atom was further confirmed by the absence of the characteristic sharp absorption peak at 8.22 ppm due to H-8 of nucleoside **2**, while the syn conformation for adenine was induced due to the bulky bromo substituent at *C*8 position (Sarma et al., 1974). 8-Alkynyl adenine nucleosides **4** were accessed, through Sonogashira cross-coupling reaction of intermediate **3** with several terminal acetylenes, under microwave irradiation (200 W) (Figure 1). In a typical experiment, an effective catalyst (Pd(PPh_3_)_4_)/co-catalyst (CuI) combination proved to be (1:1) ratio (Meneni et al., 2007) affording **4a–e**, in satisfactory yields (50–66%). To provide a detailed structure-activity relationship studies, diverse alkyne substituents R were selected which include linear alkyl chains (**4a**, R = *n*-pentyl), aromatic rings (**4b**, R = phenyl, **4c**, R = *p-tolyl)* and pyridine moieties (**4d**, R = 3-pyridyl, **4e**, R = 2-pyridyl). Finally, total deprotection of **4a–e** by the action of saturated methanolic ammonia afforded only the target derivatives **5a–d**, while attempts to remove all protecting groups from **4e** either with sodium methoxide (Bozó et al., 1998) or potassium carbonate-methanol (Plattner et al., 1972) resulted in a mixture of intractable and inseparable materials.

**Figure 2:**
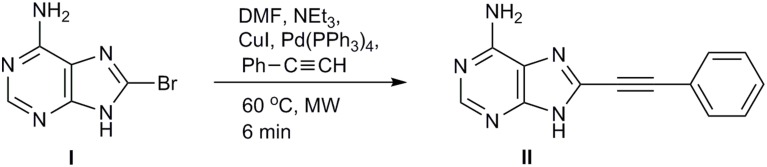
**Synthesis of 8-phenylethynyl-adenine**.

Based on the promising cytotoxic activity profile of our prior synthesized *C*5-phenylethynyl uracil glucopyranonucleoside (Dimopoulou et al., 2013) and in order to explore the impact of the glycosidic part on the biological activity as well as the potential inhibitory effects of adenine moiety, we sought to introduce phenylacetylene substituent in 8-position of adenine itself. Therefore, we investigated the development of the efficient Sonogashira alkynylation protocol for the cross-coupling of commercially available 8-bromoadenine **(I)** with phenylacetylene under microwave irradiation (200 W) (Figure 2). 8-Bromoadenine **(I)** was mixed with anhydrous DMF, phenylacetylene, triethylamine, Pd(PPh_3_)_4_, CuI, irradiated with microwaves for 6 min at 60°C and after removing volatiles *in vacuo*, the solid residue was purified by flash chromatography to provide compound **II**, in acceptable yield (40%).

### Cytostatic activity

The cytostatic activity of the novel *C*8-modified adenine pyranonucleosides **4e**, **5a–d** as well as 8-phenylethynyl-adenine **(II)** was determined against murine leukemia (L1210), human lymphocyte (CEM) and human cervix carcinoma (HeLa) cell cultures (Table 1). Compounds **4e** and **II** were less cytostatic than 5-fluorouracil (almost an order of magnitude) against murine leukemia (L1210) and human cervix carcinoma (HeLa) cells, while the same compounds proved to be more active than 5-fluorouracil against human lymphocyte (CEM) cells.

## Conclusion

In summary, we have prepared several novel *C*8-alkynyl adenine nucleosides as well as 8-phenylethynyl-adenine, *via* Sonogashira coupling conditions under microwave irradiation. Among the compound series tested, the protected adenine pyranonucleoside **4e**, as well as phenylethynyl adenine **(II)** showed significant cytotoxicity (IC_50_ of 1.2–10.0 μM) against murine leukemia (L1210), human lymphocyte (CEM) and human cervix carcinoma (HeLa) cell cultures. Since the glucose derivative of phenylethynyl adenine, nucleoside **5b**, showed no activity, it is clear that it is stable and not susceptible to hydrolysis. The replacement of glucose with ribo, arabino, and deoxyribose moieties as well as the introduction of functional substituents on the phenyl ring, such as halogens, nitro or amino and alkyl groups, could be explored in the future in an attempt to further increase the cytostatic potential of these lead compounds.

### Conflict of interest statement

The authors declare that the research was conducted in the absence of any commercial or financial relationships that could be construed as a potential conflict of interest.
